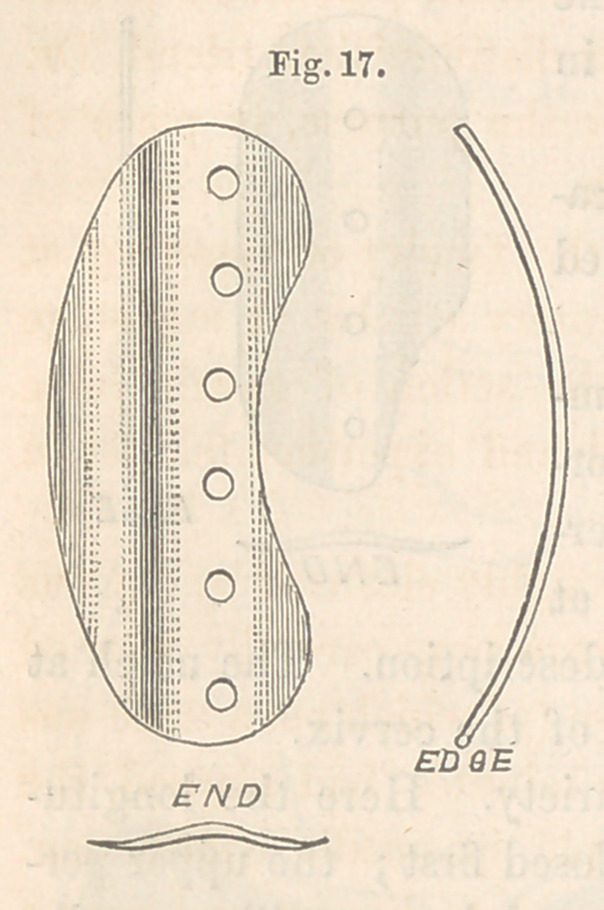# Urethro-Vaginal and Vesico-Vaginal Fistules: Their Classification and Treatment; Modifications of the Button Suture*The report of the cases, fifteen or sixteen in number, will appear in the next issue of the Review.

**Published:** 1857-07

**Authors:** N. Bozeman

**Affiliations:** Montgomery, Alabama


ART. I.—*Urethro-Vaginal and Vesico-Vaginal Fistules: Remarks upon their Peculiarities and Complications: Their Classification and Treatment: Modifications of the Button Suture: Report of Cases Successfully Treated.** By N. BOZEMAN, M.D., Montgomery, Alabama.

SOMEWHAT more than a year ago, my first paper on Vesico-Vaginal Fistule and its treatment, appeared in the Louisville Review. The views which I then endeavored to present of that disease, differ in no respect from those which had been, and are at present entertained by the profession at large; but in its treatment, I claimed to have made an important improvement, by what I termed the *Button Suture.*

Whether such a claim was based upon data sufficiently conclusive to entitle it to the credit of a place among the great modern improvements in surgery, I leave entirely to an enlightened and discriminating profession to decide.

The manner in which I was led to adopt the above mode of treatment is already before the public, and, consequently, need not be repeated here. My convictions as to its many advantages over all other methods were then, it is true, based upon a limited experience—seven successive and successful

* The report of the cases, fifteen or sixteen in number, will appear in the next issue of the Review.

operations. Still, that experience was to me doubly conclusive, as it was acquired under many disadvantages. Hence it was that I spoke positively, some may think arrogantly, of the superiority of the improvement which I conceived to have been made.

In this place, I have only to reiterate what I formerly said in favor of this form of suture. Further experience, of not only myself, but of others, fully justifies me in the importance I then attached to it.

The only change which my mind has undergone is in regard to the material of which the button should be made. In my former paper, it may be recollected that I gave the preference to silver, for the following reasons: "it is lighter, less likely to yield under pressure, admits of a higher polish, and allows the wires to be drawn through the small holes without dragging."

Admitting even now the above qualities of silver to be superior to those of lead, still, experience teaches me that they are not so essential as I formerly supposed. All these qualities, I consider, are more than counterbalanced by the softness and flexibility which lead possesses. On account of these two qualities possessed by lead, every operator, by employing that metal, is enabled to make his own buttons, and, if necessary, while his patient is on the table. When the lead is rolled out to a proper thickness, no other preparation to work it is required than a pocket knife, a punch, a small hammer, and a smooth iron plate; or, what is still better than the latter, a very small anvil, for upon this the required curves can be better formed. Very different is it with silver, to prepare which, it is necessary to employ a regular smith, who may not understand exactly what is wanted, and, even if he does, there are many little modifications in shape required which he cannot give unless the surgeon be present to point them out. Hence trouble and delay are liable to be experienced in operating, leading, perhaps, occasionally to the application of a contrivance which does not answer the purpose for which it was intended. No operator is prepared to treat the worst forms of fistule without being able, I repeat, to make his own buttons. He must always know what kind of a surface his button is to stand upon before the latter can receive the proper shape, a fact which can never be ascertained with certainty until his sutures are adjusted. Hence, again, the importance of having at hand a material out of which he can make, in a few minutes, the necessary device to meet all the indications. To this fact alone I attribute a great deal of my success in the management, as will be presently seen, of almost every variety of case which a surgeon is liable to meet with.

To what extent then has the button suture been approved by the profession, presented, as it was, upon its intrinsic merits alone? This, I think, may be partly inferred from the results obtained by its employment in the hands of other practitioners. Some of these results have already been given to the public through the journals, both of this country and Great Britain; and others have been communicated to me in a way equally reliable. The names of the surgeons who have operated according to this method, are, in our own country, Dr. Gaston,* of Columbia, S. C.; Dr. T. Wood,† of Cincinnati; Dr. Kollock,‡ of Savannah; and Dr. Williams, of this city; and, in Great Britain, Mr. Isaac Baker Brown;§ and Dr. Wm. Spencer Wells,|| the former of the St. Mary's and the latter of Samaritan Hospitals, London.

There are others, doubtless, who have employed this form of suture, but as yet their results have not come to my knowledge. I regret very much that the limits of this paper will not allow me to present the minute details of the cases reported by the above gentlemen. Suffice it to say that most of their operations were performed under unfavorable circumstances, and none, so far as I have been able to learn, excepting in one instance, required repetition. This was in the case of Dr. Williams; and judging from this gentleman's account of the difficulties which he had to contend with, the success attending even his second operation was remarkable, and not only proves the advantages of the method adopted, but reflects great credit upon Dr. Williams's skill as an operator. Several of the other cases had been previously operated upon, according to other methods.

Whether these results corroborate my former statement as to the superiority of the button suture over all other methods or not, they will, at least, show with how much certainty any one who chooses to perform the operation may look for like success.

Before referring now to the results of my own practice since the appearance of my former paper, I propose offering some remarks upon the peculiarities and complications of *Urethro-Vaginal* and *Vesico-Vaginal Fistules*, with a view to their classification and treatment by certain modifications of the button suture, now presented to the profession for the first time in a systematic form.

Urinary fistules, appertaining to the female, present great diversity of forms, as every one who is at all familiar with the subject must know. It is the rarest thing, indeed, to meet with two cases exactly alike. The urethra may, for example, be found simply cut in two, or slit at any point, or torn at its outer extremity. Again, the fistule may be situated in the trigonus vesicalis or the *bas-fond* of the bladder; in either of which regions

* MS. Letter to the Author. † Western Lancet.
‡ MS. Letter to the Author. § London Lancet.
|| Times and Gazette.

its size may vary from that of a fine probe, to that of a quarter of a dollar, or even something larger.

In the third place, it may be formed at the expense of a part or nearly the whole of the former of these regions, and the root of the urethra, or both together, or of the latter alone. Lastly, it may complicate the cervix uteri in various ways, as I shall presently point out more particularly. In all these varieties the vaginal canal suffers more or less contraction, and, as very often seen, may be divided into different compartments, or cul de sacs, by hard and unyielding bands.

A proper classification, comprising all the varieties of fistule, I consider of the greatest importance in a practical point of view. The inexperienced operator, without such an arrangement to guide him in the details of his treatment, will find his way to success beset with many difficulties, and will, consequently, often experience disappointments.

Velpeau,* in his classification, makes three divisions, as follows:

1st class embraces all those fistules "which cause a communication between the urethra and vagina."

2d class is made up of "those which are established at the expense of the trigonus vesicalis."

3d class comprises all those situated in the *bas-fond* of the bladder.

I have no objection to the classification of this illustrious author and operator, and shall adopt it so far as it goes. But there are cases, according to my experience, which cannot be referred to any one of these divisions, and, from their importance, deserve a separate consideration.

Therefore, that every case presenting itself to the surgeon may be arranged under its appropriate head, I propose to extend the classification of Velpeau, by adding two other divisions, making in all five.

The 4th class I would have to embrace all those fistules formed at the expense of a part or the whole of the vesical trigone and the root of the urethra; of the trigone and *bas-fond* of the bladder; or, all three of these regions together.

In the 5th class, I would include all those complicating the cervix uteri, either with or without injury.

As to the extent of each fistule belonging to each of these several classes, it may be very small, or it may involve nearly the whole of its respective region or regions, as may be seen by reference to some of the illustrative cuts in a subsequent part of this paper.

In cases of double or triple fistules, the openings may belong to one class alone; in any event they would be likely to be small, as is usually observed in those forms of the disease. I have myself seen two, but never three. Only one generally is referable to a class. As regards the fre-

* Operative Surg., vol. iii, p. 530.

quency with which each class is met with, an examination of twenty-four cases, enables me to give the following summary:

Of the twenty-four cases, eighteen were single, four double, and two triple, making an aggregate of thirty-two fistulous openings. Twenty-seven of these fistules were vesico-vaginal, and five urethro-vaginal. Three of the double cases were vesico-vaginal, and the others urethro-vesico-vaginal. Both triple cases were urethro-vesico-vaginal.

The whole, then, may be arranged under the following heads:

| | | |
|---|---|---|
| Single Fistules, | . . . . | 18 cases. |
| Double " | . . . . | 4 " |
| Triple " | . . . . | 2 " |
| Class 1st, | . . . . . . | 5 fistules. |
| " 2d, | . . . . . . | 9 " |
| " 3d, | . . . . . . | 6 " |
| " 4th, | . . . . . . | 3 " |
| " 5th, | . . . . . . | 9 " |

It would appear, then, from the foregoing, that fistules of the vesical trigone, and those complicating the cervix uteri, occur equally in point of frequency, and that both these classes together comprise 59 per cent., or somewhat more than one half of all. Those of the *bas-fond* of the bladder come next; then those of the urethra, which together constitute about 34 per cent., or about one-third. Lastly, it will be seen that fistules of the fourth class are the least frequent, amounting to only about 9 per cent.

Fortunately, the fourth class is rare, for I regard fistules of the first and third varieties of this class, presently to be described, as the most unfavorable forms of the disease ever met with, especially if much of the urethra has been destroyed; for in that event incontinence of urine is liable to follow, however successful an operation may be.

I might, in this connection, illustrate the peculiarities of each of these several classes with cases which have come under my own observation, but this I deem entirely unnecessary. After noticing, therefore, class first, I shall pass over the second and third with only a few remarks to a consideration of the fourth and fifth, which are infinitely the most difficult forms of the disease to understand, and the successful management of which must be viewed as one of the greatest triumphs of surgical art.

The cases to which I have just alluded as claiming our attention, present, as I shall presently show, several varieties, and to assist in a better understanding of them, and the modifications of the button required in their treatment I have introduced drawings* illustrative of each. Those

* These drawings were executed by my friend, Mr. Edward King, of this city, for whose trouble and kindness I desire here to express my sincere thanks.

intended for the former purpose were taken from a vagina with its posterior wall removed by a horizontal section. The observer is therefore supposed to be looking at the different fistulous openings from behind.

Class 1st. This class comprises all injuries of the urethra which establish a communication between it and the vaginal canal.

I say *injuries*, so as to include every variety; for there is one form of communication, at least, according to my experience, which cannot be properly termed a fistule. I refer to a rent extending from the meatus urinarius backwards, to a greater or lesser extent. The distinctions I consider of very great practical utility, and I am surprised that they should have been overlooked by M. Velpeau and other writers upon the subject. The shortening which the urethra undergoes by such an injury, is attended by some very unpleasant consequences: as, for instance, an irritable condition of the sides of the rent, and an escape of a small portion of the urine into the vulva during micturition; the fluid running down upon the thighs, and adding very much to the annoyance of the unfortunate subject.

Of the five cases belonging to this class, which have come under my observation, three were of this variety, showing that it is not of infrequent occurrence. My experience teaches me that it is the most unfavorable form of all the urethral injuries. It would be interesting to know the cause of this accident. I have inquired closely into the subject, but as yet have not been able to obtain any satisfactory information. In two of my cases, instruments had been used to effect delivery, but in the other, nothing of this sort occurred. Yet in all three cases, the nature and extent of the injury were almost identically the same, the rent being about three quarters of an inch in length. Could the awkward use of a catheter be attended by such a result? I have thought that this might be the true explanation.

Another variety of urethral injury is a mere opening or slit in the canal, which may be at its middle or near either end; and still another, where the canal is completely cut in two. This latter, I imagine, is very rare, and, when it does occur, will generally be found situated near the mouth of the canal, and is evidently produced by the parts being pinched between the child's head and the edge of the pubic bones during labor. Regarding it as a very peculiar and interesting form of the disease, I have introduced the annexed cut, Fig. 1, as an illustration of the only case I ever met with. E and F are the two ends of the urethra.

Classes 2d and 3d. These two classes, one including fistules of the trigone, and the other those of the *bas-fond* of the bladder, according to my experience, comprise about one-half of all the fistules met with in practice. They constitute the most simple forms of the disease which we

Fig. 1.

are ever called upon to treat. For this reason, and the want of space, I shall dismiss their further consideration, contenting myself with a reference to my former paper upon the subject, where all necessary information in regard to their nature and treatment may be found. The four cases there reported belong to the one or the other of these classes.*

Class 4th. Fistules of this class, as already stated, are formed at the expense of a portion of the vesical trigone, and the root of the urethra; of the former of these regions and a part of the *bas-fond* of the bladder; or of all three of these regions together. The first variety here mentioned is of rare occurrence. I have myself met with but two instances of it.

The annexed drawing, Fig. 2, is an illustration of this variety. The opening, it will be seen, involves only a small portion of the trigone, and, perhaps, not more than one-eighth of an inch of the urethra. It is oval in shape, with its longest diameter transverse, a peculiarity which will generally be found to exist. The posterior edge, soft and yielding, can, in nearly if not quite all of such cases, be brought down so as to close the opening without disturbing the anterior edge, which latter is always immovably fixed to the pubic bones. In addition to this peculiarity of the anterior edge, there is another, sometimes met with, which has a very important bearing upon any operative procedure that may be adopted. I allude to its inversion, in which case coaptation of the two edges is rendered impossible, and the action of the sphincter vesicæ, if any remains, must, to some extent at least, be destroyed. Cases presenting this feature cannot, therefore, be regarded otherwise than unfavorable. Of the second variety of this class, I have not seen a single instance. When it occurs, as most unquestionably it does sometimes, I would regard it almost as favorable

* See Louisville Review, May, 1856.

as the varieties of the two preceding classes. Both borders of the opening would be likely to prove yielding and quite susceptible of mutual approximation.

Fig. 2.

The third variety I have met with but once, and, considering the nature and extent of the injury, it must be looked upon as by far the most unfavorable form of fistule ever presented to the surgeon. Not but what it is just as readily closed as some of the other forms. The idea I wish to convey is, that the result of the operation will be incomplete, owing to so much of the root of the urethra, and the trigone, and *bas-fond* of the bladder having been destroyed.

In this variety, too, we are almost certain to find the same peculiarity of the anterior edge pointed out in connection with the first,—namely, inversion. This, of course, always adds to the unfavorableness of the case; and upon the existence of this feature I have based an operative proceedure which, so far as my information extends, has never before been employed by any one else. This will be described in its appropriate place.

The annexed cut, Fig. 3, is a view of the fistulous opening in the case last mentioned. It is a most excellent type of this variety in its very worst form. The opening is quite large, measuring one inch and seven-eighths transversely, and one inch and three-eighths longitudinally. The dotted line represents the inverted border, and X the beginning of the urethra.

Class 5th. This class embraces all fistules, of whatever shape or size, to which the cervix uteri, whether itself injured or not, bears close and important relations. I have stated that about 29 per cent. of all the cases met with in practice belong to this class. The varieties which it presents are several, and from their peculiarities and importance, claim our special attention. These I shall attempt to point out as precisely as possible, as I go along, for upon them is based some of my most valuable modifications in the plan of treatment by the button suture.

First, the fistulous opening may be found extending just across the tip of the anterior lip of the cervix uteri, which latter forms its posterior border. It may be barely large enough to admit the index finger, or it may involve nearly the whole of the *bas-fond* of the bladder. The accompanying cut, Fig. 4, is an illustration of the latter extreme of this variety. I have met with two cases of this description.

Fig. 3.

Fig. 4.

Secondly. Nearly the whole of the *bas-fond* and trigone of the bladder may be implicated, and in some instances, indeed, even the root of the urethra. The former variety I have seen, but not the latter. In my case, the whole of the space between the cervix uteri and mouth of the urethra had sloughed out, the destructive process involving the extremities of both ureters. The opening measured one inch and five-eighths transversely, and one inch and three-eighths longitudinally. The accompanying figure, 5, represents the size and shape of the fistule, and the points related to it. D, is the mouth of the urethra; B, the left ureter; and C, the right. The last, it will be seen, is represented as opening on the vaginal side of the septum. This condition really existed, and resulted from an eversion of the edge of the fistule at this point. In addition to the above, the anterior border was immovably fixed to the pubic arch.

Thirdly. The fistule may present the appearance of a longitudinal or oblique slit through the vesico-vaginal septum, complicated with a rent in the anterior lip of the cervix. The rent may be in the centre, or at one

Fig. 5.

Fig. 6.

corner of the lip, and the line of its axis may or may not correspond to that of the fistule. In the latter form, the axes of the two, I imagine, never correspond. The accompanying drawing, Fig. 6, represents a longitudinal fistule complicating the anterior lip at its centre. Fistules of this variety are generally formed without much loss to the vesico-vaginal septum; differing widely in this respect from the other varieties belonging to this class. I have met with both these varieties.

Fourthly. Whether the fistule be longitudinal or transverse, small or large, the cervix is not complicated with it in the form of a simple rent, but a part or the whole of that portion included within the vagina, or

Fig. 7.

Fig. 8.

even more, may have been destroyed, with or without closure of its canal. It is this form of injury which M. Jobert claims to have been the first to describe under the name of Vesico-Utero-Vaginal Fistule.

The accompanying sketch, Fig. 7, of a case now under my charge, illustrates one form of this injury, in which there is a loss of the cervix. The fistule in this case, as will be seen, is of a peculiar shape. Its upper part (defined in the figure by a dotted line carried across the fissure), is longitudinal, and formed at the expense of the vesico-vaginal septum; while the lower is transverse, and corresponds to that part of the cervix destroyed. A, marks the relative position of the uterine orifice. Another sketch, Fig. 8, of a case which I have just cured, shows the entire loss of the vaginal portion of the cervix and the vaginal attachment to its anterior aspect. The cervical canal, so far as I have yet been able to ascertain, is completely closed. Not even a depression exists to indicate its whereabouts.

This complicated fistule, as will be seen, is nearly circular, and includes almost the whole of the *bas-fond*, and a part of the trigone of the bladder. The cases which these two last drawings illustrate, come under the head of vesico-utero-vaginal fistules, according to Jobert, and are the only ones I have ever met with.

As to the other form of cervical injury, described by the above surgeon under the head of *Vesico-*

*Uterine Fistule*, I have yet to see a case, and as it has not presented itself once in twenty-four cases, I infer that it must be exceedingly rare. Indeed it is difficult for me to conceive how a communication could be established between the bladder and the cervical canal without implicating the vagina. From my knowledge of the anatomical relation of the parts, I know that such a thing is possible; but the question is, could a cause be brought to bear upon a space so contracted as to produce the result? If Velpeau's account of this form of injury is correct, then Jobert makes a distinction without a difference between it and vesico-utero-vaginal fistule. In speaking of Jobert's first case, he says: "The anterior wall of the neck of the uterus, and the corresponding portion of the bladder were entirely destroyed."* Now I ask how could the anterior wall of the cervix be entirely destroyed without involving the vagina? Without discussing this point further, I am forced to the belief that even Jobert himself never met with a case which could be appropriately called vesico-uterine fistule,—that is, if Velpeau's interpretation of his views are correct. However, whether one or both of these forms of cervical injury are appropriately named or not, is of but little consequence in a practical point of view. They appear to me to be liable to cause confusion, without, in the least degree, aiding in a proper understanding of the subject. For this reason, if no other, I shall consider all the varieties of fistule belonging to the class now under consideration, under the head of Vesico-Vaginal, and the different injuries of the cervix uteri as mere complications, which do not deserve to be regarded in any other light.

*Treatment.*—I shall call attention first to the management of rents at the outer extremity of the urethra, and for reasons such as those given in a previous allusion to the second and third classes, shall then pass on to a consideration of the special treatment applicable to the different varieties of the fourth and fifth classes.

I have already mentioned that the variety of urethral injury above referred to has hitherto been almost, if not entirely neglected by writers upon the subject. Of course, therefore, no special plan of treatment, at least none sanctioned by experience, is to be found upon the records of the profession. Velpeau, in speaking of a case which he had at La Charité, in which the fistule was situated near the extremity of the urethra, says that, "he confined himself to the extirpation of the urethral bridle which separated the fistule from the meatus urinarius."† By this procedure he produced the very same form of injury, the treatment of which I am now about to describe. As a preliminary step, this course of Velpeau's may, under some circumstances, be justifiable; but does our obligation to the patient cease with

* Op. cit. vol. iii, p. 872, Am. Appendix, by G. C. Blackman, M.D.

† Op. cit. vol. iii, p. 530.

this? It would really seem so from a further remark of this surgeon. For, says he, "this small operation was attended with entire success." Now if fistules of this class, because they are near the meatus urinarius, are to be treated in this way, without anything further being done, it does seem to me that such surgical interference as the above is worse than useless; for by it we convert a simple form of injury into one which will more severely tax the patience of the surgeon to effect a complete cure than almost any of the other varieties pointed out as belonging to this or the two preceding classes. A plan of treatment then, adapted to this form of injury, is, I maintain, of the utmost importance. Whether the comfort of the patient is sufficient inducement for its employment or not, the credit of our art requires that it should, at least, be placed upon a sure basis.

The following procedure, which I am in the habit of practising, will be found simple, easy to perform, and, according to my experience, well-suited to the purpose.

First, the sides of the cleft should be thoroughly pared, and sutures introduced, and the parts brought together as in any other variety of fistule. But in doing this a very important point is to be borne in mind, viz., getting rid of the action of the urine as it passes through the newly formed channel. This must always be attended to if we expect to succeed. As a protection to the parts, therefore, a catheter would very naturally suggest itself, and I consider this the only means that could be devised. But this alone is not sufficient to insure a perfect result, however well the operation may be performed,—and why? Because the instrument, the very means employed to ward off one difficulty, becomes itself an impediment to union of the parts by the first intention. This impediment is caused by the weight of the instrument, and cannot well be prevented, no matter what form or variety be employed.

Fig. 9.

There being no support to the catheter at the meatus, its constant tendency is to tilt downwards between the two denuded edges of the cleft, however well they may have been brought together. Partial or complete failure will, therefore, be the inevitable result. Now, how is this support to be obtained? Certainly, not by attaching a contrivance to the body of the patient, or to the couch upon which she is lying; for the slightest movement of the former would derange the whole affair. The only way in which I have been able to attain the desired end is by a modification of the button, which I employ in cases of vesico-vaginal fistule. The accompanying sketch, Fig. 9, is a representation of such a contrivance. The usual shape of the button is preserved, with the addition of the notch in the end and the curve indicated in the side view. It is also a little narrower than the common button, being about half an inch in width. The curve is made to correspond to the general direction of the urethra. When this contrivance is applied and secured in its place by compressing shot upon the several sutures, as is usually done, the end with the notch projects forwards, and in front of the meatus urinarius, and thus becomes a stationary point upon which the catheter may rest, without in the least interfering with the denuded edges of the cleft. The catheter should always be introduced before the button is secured in its place, and not removed, if it can possibly be avoided, until the cure is complete; nine or ten days generally sufficing. When it chokes up, as sometimes occurs, it can be easily opened by running a small wire through it. When the suture apparatus is removed the catheter should be cleansed, replaced, and worn for three or four days longer. The pressure may now be taken off from the tender cicatrix and the instrument held sufficiently steady by means of a loop attached to a belt, carried around the body of the patient. If this precaution be not observed, and the catheter be allowed to hang down, the rent is almost certain to be reproduced, either partially or completely. I have known the latter to happen two weeks after the cure was thought to be complete, in an instance where the catheter was worn after an operation for vesico-vaginal fistule.

The catheter which I have found to answer best in this operation, is the male elastic, of English manufacture. No. 5 is about the right size. I use it of full length, because it is more comfortable to the patient; and the danger of any urine running up on the outside to the denuded edges, is more effectually guarded against. I have several times performed this operation, and always with the happiest results.

In two of my cases, the rent was associated with double vesico-vaginal fistules; in the other, with a single one. In the first two, I attempted closure of the rent and one fistule at the same operation; in each, the rent closed, but not the fistule. Judging, therefore, from these results, I think it best always to make separate operations of the two forms of injury. This will be my course hereafter, and I think it preferable to close the fistule first, for the reason that the cure of both can be completed sooner, and no danger result to the urethra from the pressure of the catheter, as there would otherwise be if the latter was operated upon first. The after-treatment of the two together cannot be made to harmonize, and here, I am satisfied, lies the cause of my failure to close the fistules in the two cases referred to. The catheter, when constantly worn, as it should be for the rent, is very liable to get closed up, and when it is not promptly attended to, mischief to the fistule is certain to follow from an accumulation of urine in the bladder.

*Fourth Class.* In calling attention to the management of this class, I shall confine myself to the first and third varieties. The second variety is simple, and requires no modification in the ordinary plan of treatment.

One form of treatment applicable to the varieties referred to, is also simple, and does not require a protracted notice. The only peculiarity about the operation is dependent upon the fact that the anterior border of the fistule is immovably fixed to the pubic bones, and when the edges are pared and the sutures introduced, it remains *in situ*, while the posterior is hauled down, causing sometimes a slight depression of the uterus. When the edges are thus approximated by the adjustment of the sutures, the surface upon which the button is to stand may be found either convex, from previous thickening of the urethra, or concave, from close attachment of the anterior border to the pubic bones. In the former case, a button bent upon its concavity will be required. Fig. 10 is a front, edge, and end view of such a one as I have recently employed. When the surface is concave, the button will require to be bent upon its convexity, as represented in Fig. 11.

Fig. 10.

Fig. 11.

There is, however, another form of treatment sometimes applicable to these two varieties, which is a little more difficult to carry out. In addition to the modifications of the button, which are the same, there is a difference in the plan of paring the edges of the fistule and the introduction of the sutures. This is required from an inversion of the anterior border, which feature I pointed out in the description of these varieties. Coaptation of the two edges is here almost impossible, and cannot be effected without detaching the parts from the pubic bones. Rather, therefore, than take this risk, I have devised a plan of operating, to allow the inverted border to remain undisturbed. The procedure is as follows:

The posterior edge, instead of being bevelled, is in this instance to be pared square, or, in other words, perpendicularly, around to each commissure of the fistule. At these two points, the process of denudation, instead of being carried along the anterior edge, is continued parallel to it across the anterior wall of the vagina, and the raw surface made of sufficient width to match the posterior edge. Fig. 3 illustrates a fistule of this character, with its edges pared. The circular lines, as will be seen by reference to the cut, show the situation and extent of the denudation upon the anterior vaginal wall.

The paring being completed, sutures (always equal numbers upon the two sides of the urethra) are next introduced. The peculiarity connected with this step, is that the vaginal mucous membrane is pierced three times by the needle in lodging each suture in its respective place. For example, the needle is entered just in front of the denuded surface, is carried deep into the substance of the vagina, and then brought out just behind the same denuded surface. Next, it is carried across the fistule and through the opposite edge in the usual way; and so on until the requisite number, varying from two to eight, is introduced. This being done, the sutures are to be adjusted in the ordinary manner, which causes the posterior edge to rest upon the anterior vaginal surface. The surface upon which the button is to stand, will, almost if not quite always, be found concave. Therefore, such a shaped button as Fig. 11 illustrates, will be required. The length and extent of the curve will always depend upon the length of the axis of the fistule. Fig. 12 is a view of such a button as was employed in the case represented by Fig. 3. One longer and having a greater curve than this will, I imagine, be rarely if ever called for. The object of the curve in all cases is to bring the two ends of the button within the pubic arch, the only way in which perfect adjustment can be had.

Fig. 12.

*Fifth Class.*—After the account already given of this class of cases, it would be a waste of time to dwell on the difficulties and dangers which have heretofore been regarded as inseparable from any operative procedures intended for their management. It may not be deemed out of place, however, to refer briefly to the course pursued by some surgeons of good reputation.

Vidal (de Cassis), has declared that fistules, even of the *bas-fond* of the bladder, due to a loss of substance, are entirely beyond the reach of art, so far as their closure is concerned. He would, of course, in this, as he did in that class of cases, advise obliteration of the vagina. Now that this procedure is ever justifiable, under any circumstances, my observation and experience has yet to prove. My present conviction is, that it is rarely if ever called for. Jobert (de Lamballe), however, be it said to the honor of French surgery, has done something more. He has not only devised a plan of treatment to reach this extreme and much-to-be-lamented class of sufferers, but has actually demonstrated its utility. To this surgeon, so distinguished for his skilful exploits in the field of operative surgery, is therefore due the credit of having been the first, indeed, I may say the only one, either in this country or Europe, to accomplish anything in the treatment of the different varieties of fistule complicating the cervix uteri. His method of operating is denominated *Cystoplasty*. To this procedure, I wish now to invite attention.

The operation of cystoplasty, though ingenious, and doubtless one of the greatest triumphs of modern surgery, appears to me to be environed by some objections. These I propose to examine here, with the view of ascertaining whether the operation can ever be brought into general use or not. In attempting this, however, I do not wish it to be understood that it is my purpose to detract from the claims of its distinguished author. Far be this from me. I am, in common with the whole profession, willing to accord to him the praise he so justly deserves for his wonderful success in this hitherto unexplored branch of obstetrical surgery.

What, then, is cystoplasty? What its advantages? What the amount of skill required to perform it? What the dangers attending it? In short, what are the inducements it holds out for general adoption?

Cystoplasty, in the most extended acceptation of the term, means, I believe, the process of closing any kind of fistulous opening into the bladder. Jobert, however, restricts it to the procedure, adopted by himself, for closing openings in the vesico-vaginal septum, occasioned by extensive loss of substance. The steps of the procedure, according to my understanding of it, are as follows:

The cervix uteri is first seized with a pair of Museaux's forceps and drawn down to the vulva. Next, the vagina is severed from its anterior attachment, which brings to view the point at which the posterior wall of the bladder is connected to the cervix by areolar tissue. From this point back to the reflection of the peritoneum, a clean dissection of the vesical wall from the cervix is made. This being done, the borders of the fistule are freshened, the sutures introduced, and approximation then effected. The object of the above dissection, it will be understood, is simply to procure the requisite amount of relaxation of the posterior wall of the bladder to admit of its being drawn down so as to close the fistulous opening. In this way displacement of the uterus is said to be avoided, and consequently dragging upon the sutures prevented.

Admitting, now, the attainment of all that is here proposed, and the result to be generally satisfactory, I ask, are not objections to the procedure apparent to every one who is at all familiar with the anatomical relations of the parts? The objections which I find are:

1st. The operation has neither simplicity nor certainty to recommend it.

2d. It is unavoidably tedious and painful.

3d. A greater amount of skill is required for the necessary dissections, than falls to the lot of many who may be called upon to perform the operation.

4th. The peritoneum is liable to be injured, against which risk no amount of anatomical knowledge, skill, or dexterity in the use of instruments is a sure guarantee.

The last mentioned, I regard as the greatest objection to this mode of operating. That Jobert himself has lost patients from the inevitable result, peritonitis, there can be no doubt. Only a few months ago I saw in a letter from a Paris correspondent of one of our medical journals, that this surgeon had a short time before lost a patient from this cause after one of his operations.

That a procedure, therefore, if not more successful, at least less liable to the above objections, is a desideratum, all must admit.

My experience in the management of the unfortunate class of cases of which we are now speaking, is comparatively limited. Still, sufficient, I trust, has been acquired to justify me in presenting my views here in a matured form.

The procedure which I have adopted, and propose here to give to the profession, will, I think, be found simple, easy to perform, and last, though not least, of all other considerations, almost unattended with danger. I say it is almost unattended with danger, for I have never seen, excepting in one or two instances, the slightest accident result from my operations, which now amount to seven.

Somewhat more than two years ago, I met with my first case of vesico-vaginal fistule complicated with a rent in the anterior lip of the cervix uteri. Then it was that I first demonstrated the practicability and safety of paring the cervix, and lodging sutures in its substance. An account of this case is to be found in the Southern Medical and Surgical Journal for August, 1855.

The importance of this method of treatment, and its general applicability, I did not then fully appreciate. Indeed, I was not aware at the time, that the same thing had not been practised by other operators, and thought but little of it. A further examination, however, satisfied me that the plan was new; or at least I could find no mention of it in a diligent search through all the writings appertaining to the subject that I could lay my hands upon. Since that time, Dr. J. M. Sims,* of the Women's Hospital, New York, has adopted the method, and if I am correctly informed, others have done the same.

The value of this discovery I now consider incalculable, and, in connection with another principle of my procedure, to which I shall now call attention, enables me to cure cases with ease to myself and safety to patients, which have heretofore been abandoned entirely, or reached only by the dangerous procedure of Jobert. The principle to which I have just alluded, is the depression of the uterus for the purpose of closing the fistulous opening however large it may be. This point, although suggested by Velpeau, has never before, that I am aware of, become established as a practice by any one, and, as would appear from the language of that distinguished surgeon, he himself attached very little importance to it. In speaking of it, he says: "Another process which might be borrowed from anaplasty, when the fistule is very high up, would consist in actively cauterizing its vaginal region, then in hooking the neck of the uterus with an érigne or a noose of thread, in order to pull it down, and cause it to slide, as a drawer, below the vesical opening. But I repeat, all these suggestions want a foundation to rest upon; none of them can yet adduce any success in their favor."†

The idea contained in the above was certainly a good one, but unfortunately it was never carried out in practice, nor could it have been done successfully without a knowledge of paring and suturizing the cervix.

The principle then upon which my procedure is based, is the subserviency of the uterus for closing the fistulous opening, and the lodgment of sutures in the cervix uteri, as in any part of the vesico-vaginal septum.

If there is no danger in paring the cervix and carrying sutures through it, the only other objections which could be urged against the procedure are displacement of the uterus, and a supposed dragging upon the sutures. As to the displacement, I myself attach no importance to it. I have never yet seen any ill consequences from it, save a little soreness across the lower part of the abdomen for a few days after the operation. In a very short time, the organ, owing to the yielding nature of the vagina, ascends almost, if not entirely, to its normal position in the pelvis. In one case upon which I operated, the uterus was dragged down nearly an inch and a half, and secured to the inferior border of the fistule. The

* MS. Letter to the author. † Op. cit. vol. i, 627.

operation was entirely successful, and now, if one should make an examination of the parts without a knowledge of what had been done, he would scarcely be able to detect any alteration save a less degree of prominence of the cervix. This patient now enjoys perfect health, menstruates regularly, and is generally able to retain her urine all night.

As to the dragging upon the sutures, I fear nothing from this. I have never had a suture to cut out, and I am satisfied that, owing to the dense and firm structure of the cervix, this accident is much less apt to occur here than in the vesico-vaginal septum.

I beg leave now to call attention to the modifications of my plan of treatment applicable to the several varieties constituting this class.

Fig. 4 is an illustration of the first variety. A reference to the cut again, will prepare the mind for a proper understanding of the adjustment of the suture apparatus. The edges of the fistule, it will be seen, although I did not before call attention to the fact, are represented as being pared; and in paring the cervix, I may observe here, that it is proper always to leave the denuded surface perpendicular, not oblique or bevelled, as is required generally for the vesico-vaginal septum. This is necessary to insure a good coaptation with the opposite edge.

The paring being completed, the next step consists in the introduction of the sutures, of which two are usually required for the cervix; and these two are of the greatest importance. The number of the others will, of course, depend upon the size of the fistule; and in their introduction, the ordinary plan is to be pursued; but it is essential that those in the cervix should be attended to first, then the others upon either side. In this variety of fistule, both edges being movable, meet, when approximated, upon half-way ground.

Fig. 13 illustrates the shape of the button required in this variety, and is a representation of the one used in the case referred to. The notch in the upper edge is for the accommodation of the anterior lip of the cervix. There is nothing peculiar in the method of applying the button and securing it in its place, nor in the after-treatment.

Fig. 13.

Of the second variety, it will be recollected that Fig. 5 is an illustration. The process of paring here is the same as in the preceding variety; but the two succeeding stages differ in the introduction and adjustment of the sutures;—and why? Because the anterior border of the fistule is immovably fixed to the pubic arch, which prevents their mutual approximation. In this state of things, we may avail ourselves of that principle of independent action ascribed to the sutures required to be entered in this way. Instead of being parallel to each other, they are made to represent parts of the radii of a circle, whose centre is fixed above the uterine orifice, to a greater or less distance, according to the size of the fistulous opening. The sutures all being introduced, they are next adjusted, as directed in the preceding variety, which brings the parts together as shown in Fig. 14, illustrating this stage of the operation in the case above referred to. The

Fig. 14.

Fig. 15.

dotted lines above show the extent to which the uterus was hauled down to close the opening.

The next cut, Fig. 15, is a front, edge, and end view of such a button as was required for the parts as above arranged, and is such as will generally be found indicated in this variety.

Of the third variety, a reference to Fig. 6 will indicate what is to be done. Here we have a simple longitudinal slit complicated with a rent in the centre of the anterior lip of the cervix uteri. The steps are, first, to pare the edges of the fistule in the ordinary way, and then the two sides of the cleft; next introduce a sufficient number of sutures to close, first, the fistule, and then the cervix; only one or two being generally required for the latter. The sutures for the cervix, in this instance, are introduced transversely, instead of antero-posteriorly as in the preceding varieties. The whole being adjusted, the sides of both fistule and cleft are thus brought in apposition.

Fig. 16.

Nothing more now is required but the application of a button, whose shape must be fashioned to suit the parts upon which it is to stand.

The oblique form of the fistule, sometimes, complicating the lip of the cervix at one of its corners, had better be operated upon alone; afterwards the rent. Such a button as represented at Fig. 16, will be found suited for fistules of this description. The notch at the corner is for the accommodation of the side of the cervix.

Fourth variety.—Fig. 7 is a view of this variety. Here the longitudinal or lower portion of the fistule should be closed first; the upper portion, at a subsequent operation. A common-shaped button will generally be found to answer, no modification of any importance being necessary.

In closing the upper portion of the fistule, however, a little more trouble will be experienced. The loss of a portion of the cervix, and the anteversion which sometimes attends it, are both difficulties to be encountered. If the latter condition does not exist, the same treatment applicable to the first variety will answer, the only difference being that the button will not require to be so long. If anteversion of the cervix, however, does exist, then the process of paring and introducing the sutures must be varied; that is, if it is desired to exclude the uterine orifice from the cavity of the bladder, which should always be done if possible. In this instance, the paring is done on the anterior edge of the remaining portion of the cervix, and, as a matter of course, involves a portion of the vesical mucous membrane; this is necessary to obtain a sufficiently broad surface anterior to the os uteri to insure agglutination of the two edges when brought together. Next, the sutures for the cervix should be entered far in upon its vesical side, and brought out from behind forwards, on a line that will allow of coaptation of the denuded surfaces without interfering with the uterine orifice. Traction now made upon these sutures, while it carries the anterior edge up, at the same time brings the cervix downwards and backwards towards its normal situation, and thereby forces the os uteri to a position posterior to the line of approximation. In this way the catamenia will be allowed to take the natural outlet instead of through the bladder, as would necessarily be the case were the fistule closed with the cervix *in situ*. A button of the shape last referred to, will be found to answer here also.

The remaining form of fistule belonging to this variety is seen at Fig. 8. Here, although the limits of the cervix are not well defined, still, the remnant of it forms the posterior border of the fistule, and must be pared along with the other parts of the fistulous border. This and the introduction of the sutures are done very much in the same way pointed out for cases belonging to the first variety. The principal difference being that the stump, so to speak, of the cervix is broader, and consequently will hold a greater number of sutures. Owing to this difference, the notch in the upper edge of the button will also require to be proportionately larger. The accompanying cut, Fig. 17, is a view of such a one as I have used. In addition to the notch being a little larger, the button is slightly bent upon its concavity, which peculiarity is required for easy adjustment. The edge view indicates the extent of the latter.

## Figures and Tables

**Fig. 1. f1:**
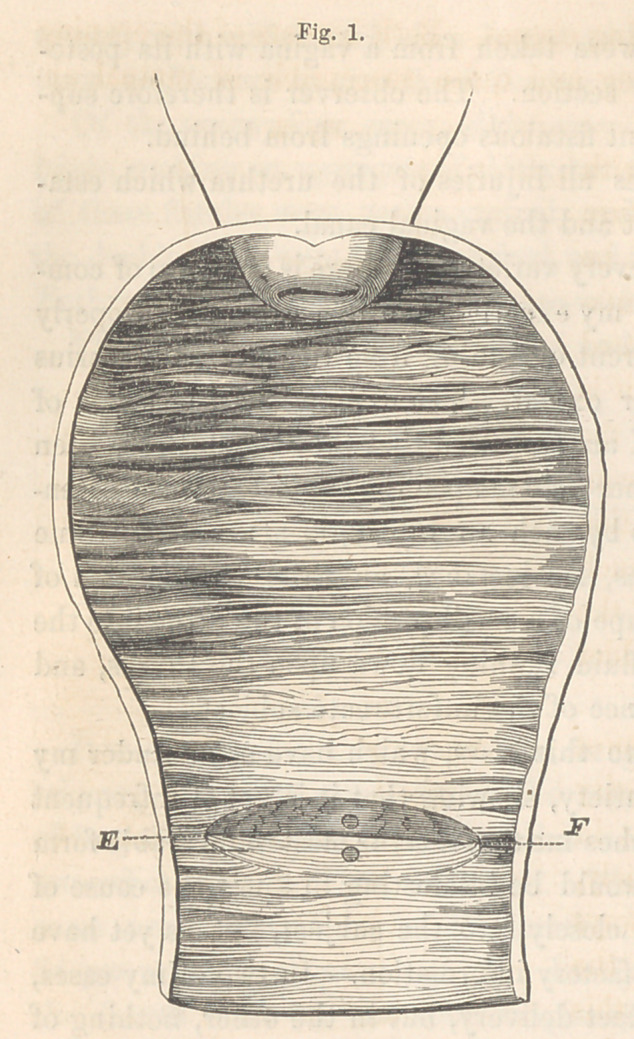


**Fig. 2. f2:**
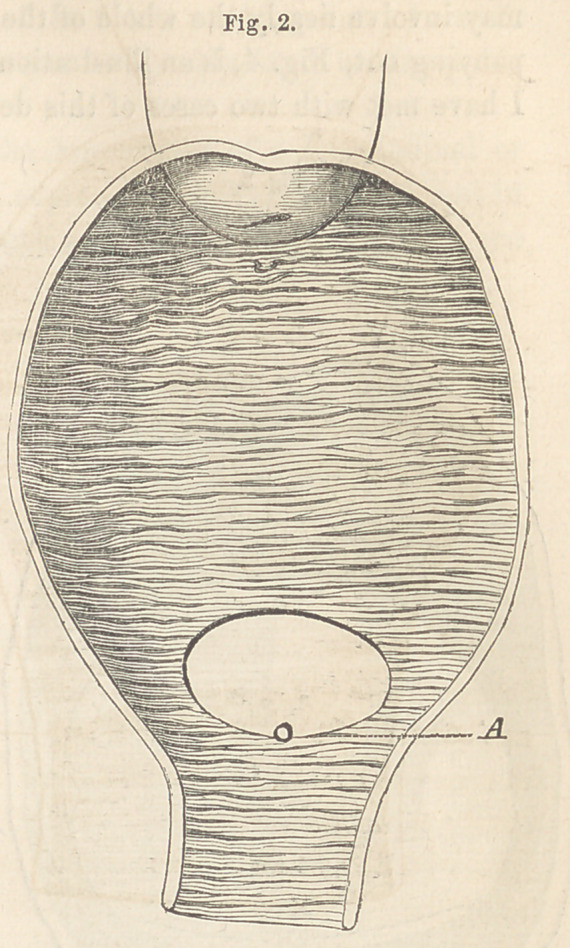


**Fig. 3. f3:**
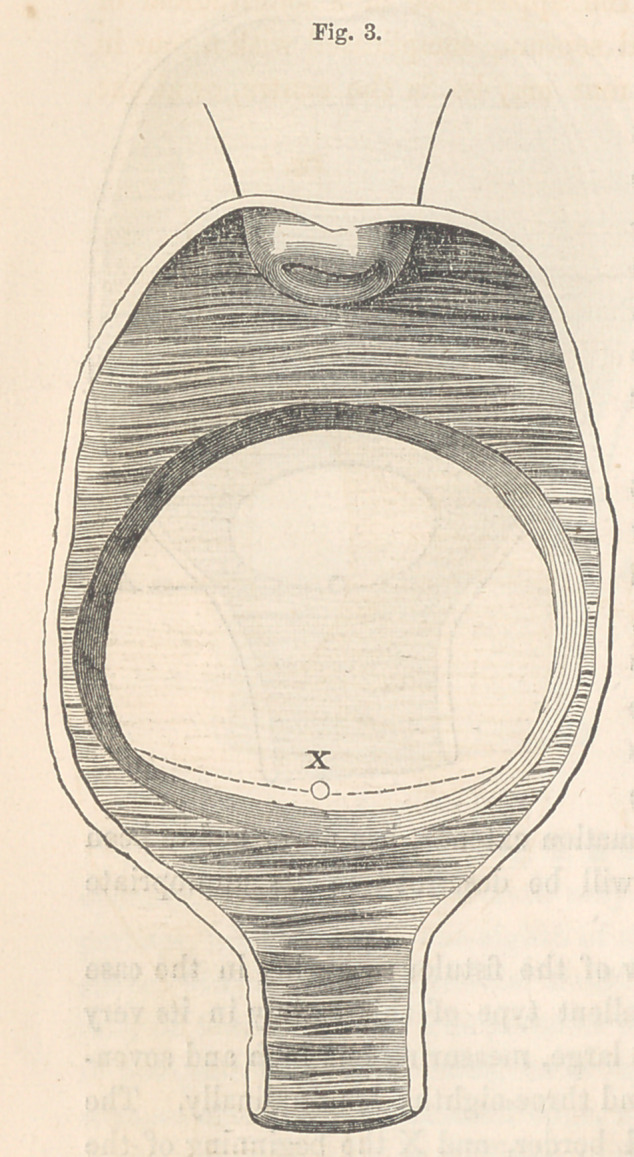


**Fig. 4. f4:**
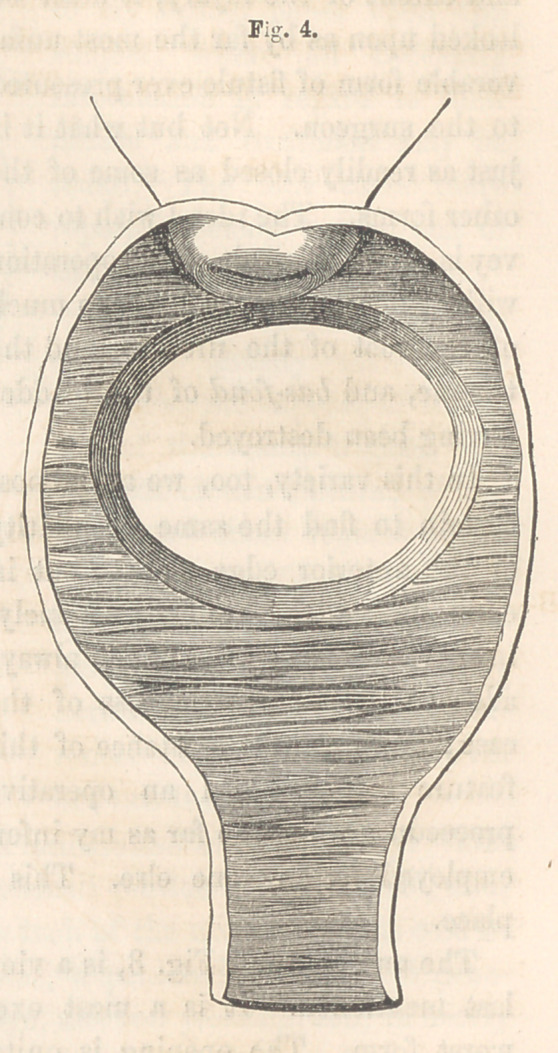


**Fig. 5. f5:**
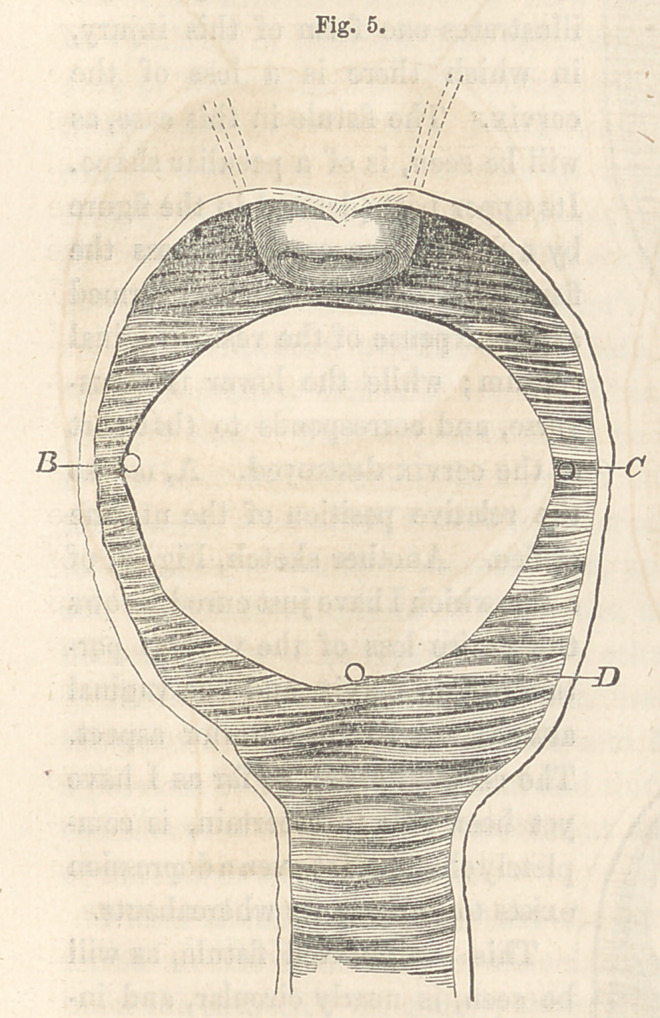


**Fig. 6. f6:**
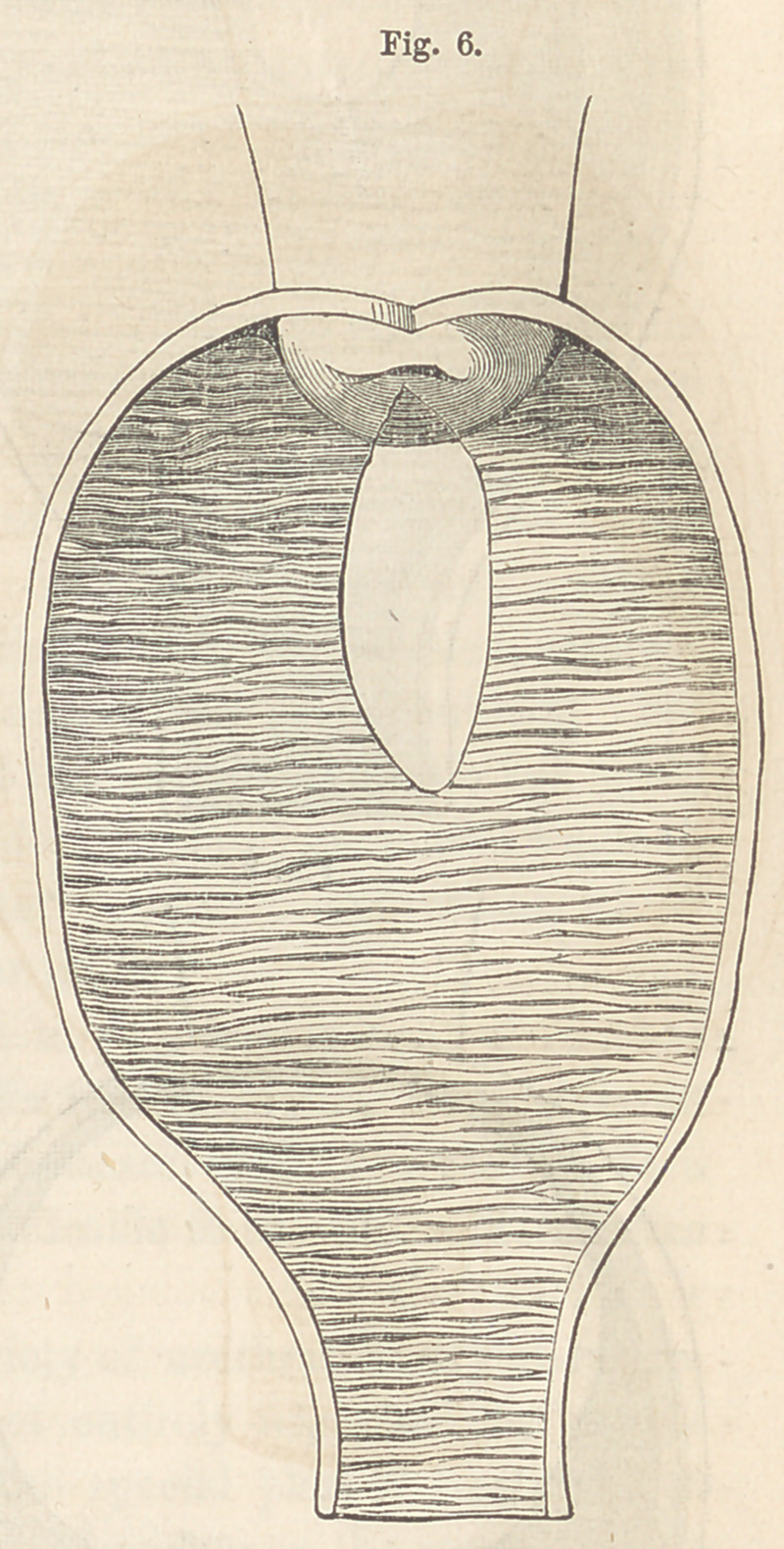


**Fig. 7. f7:**
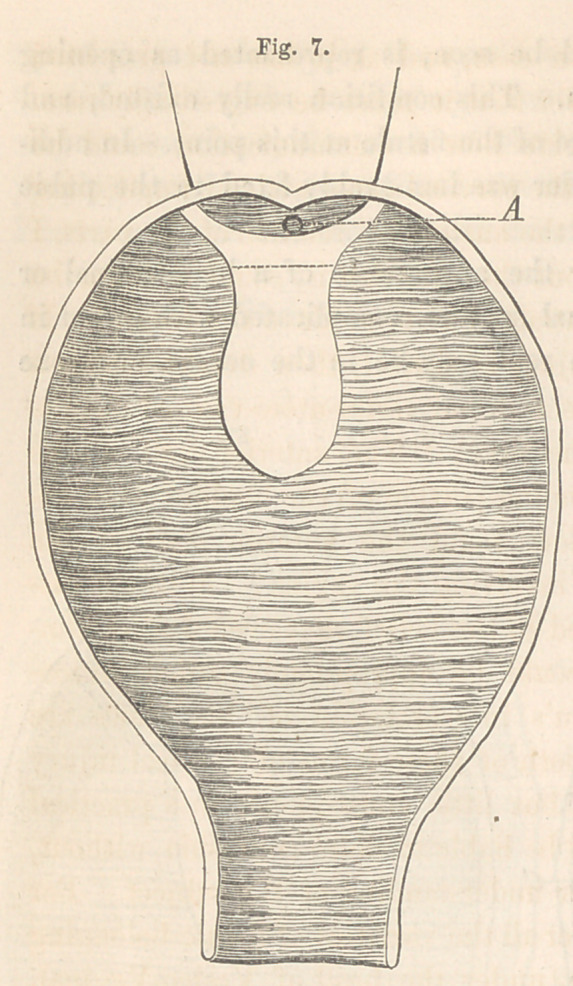


**Fig. 8. f8:**
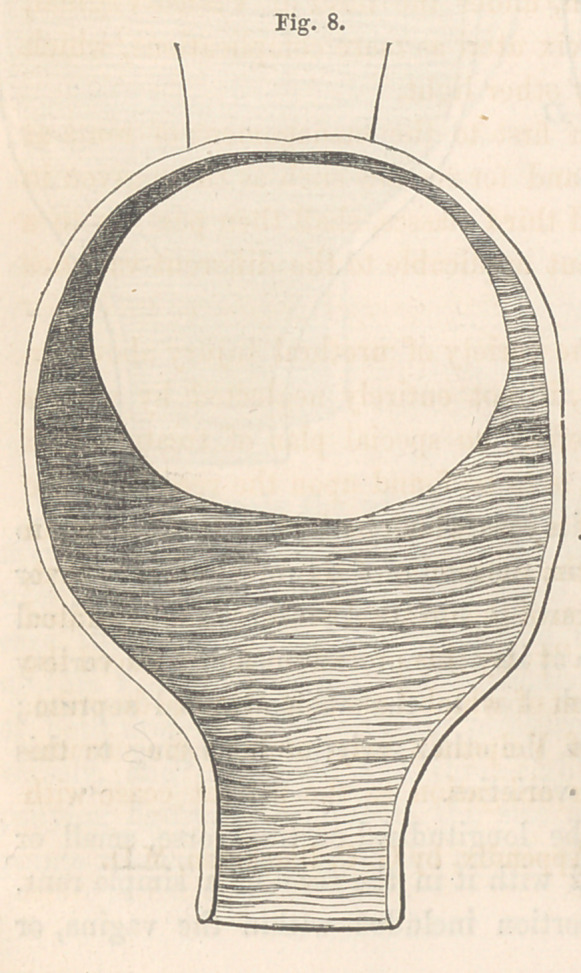


**Fig. 9. f9:**
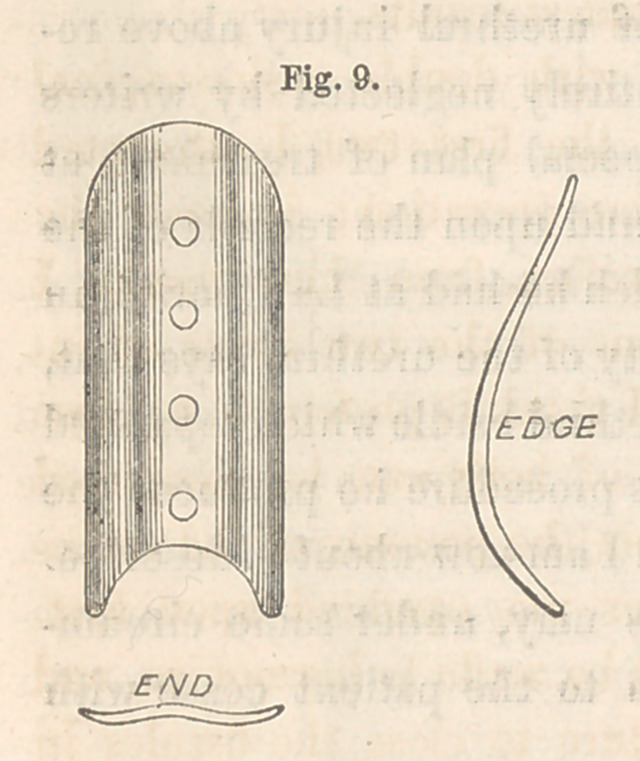


**Fig. 10. f10:**
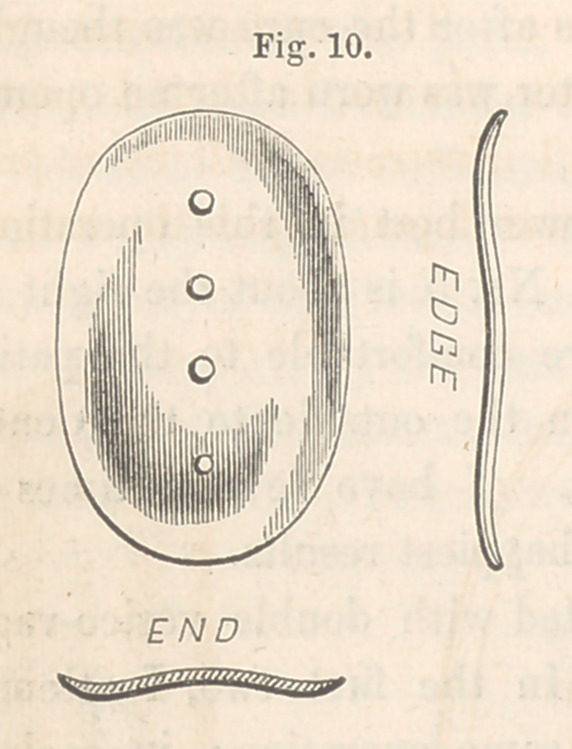


**Fig. 11. f11:**
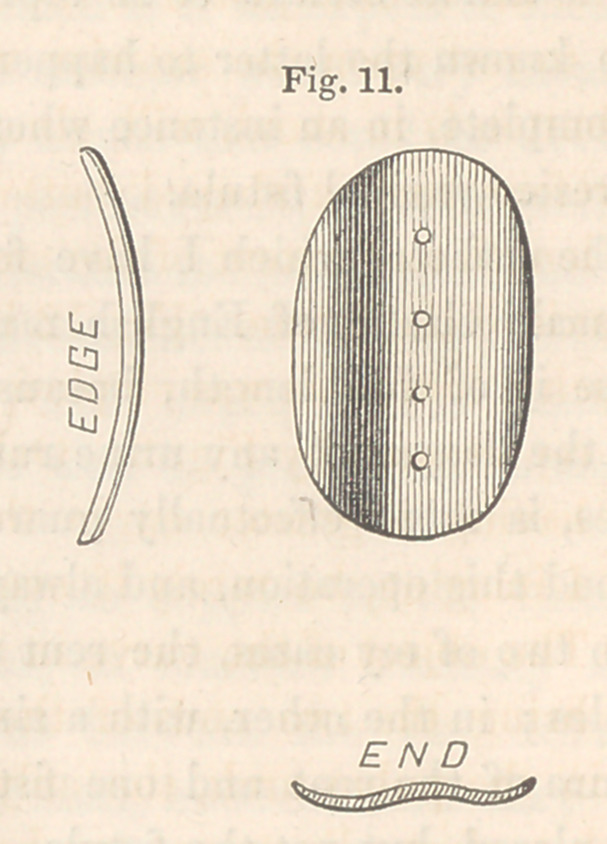


**Fig. 12. f12:**
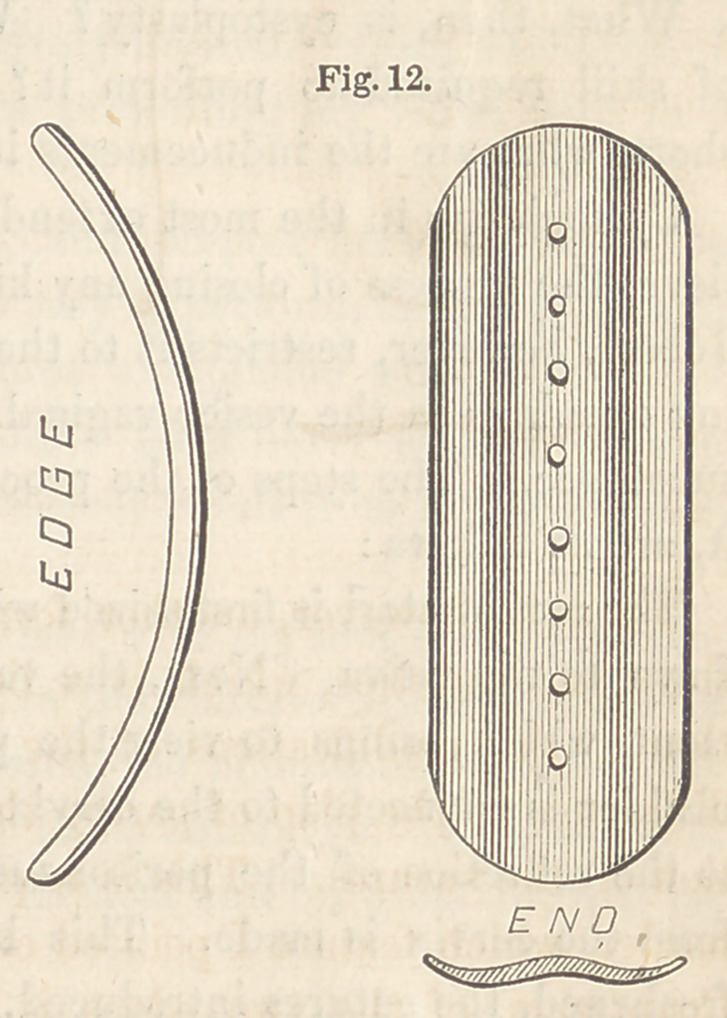


**Fig. 13. f13:**
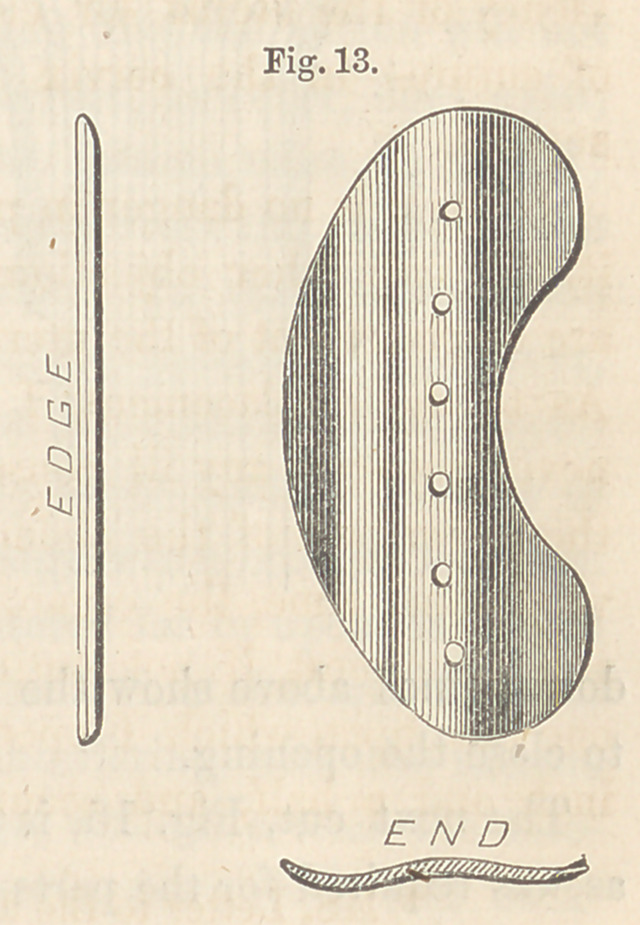


**Fig. 14. f14:**
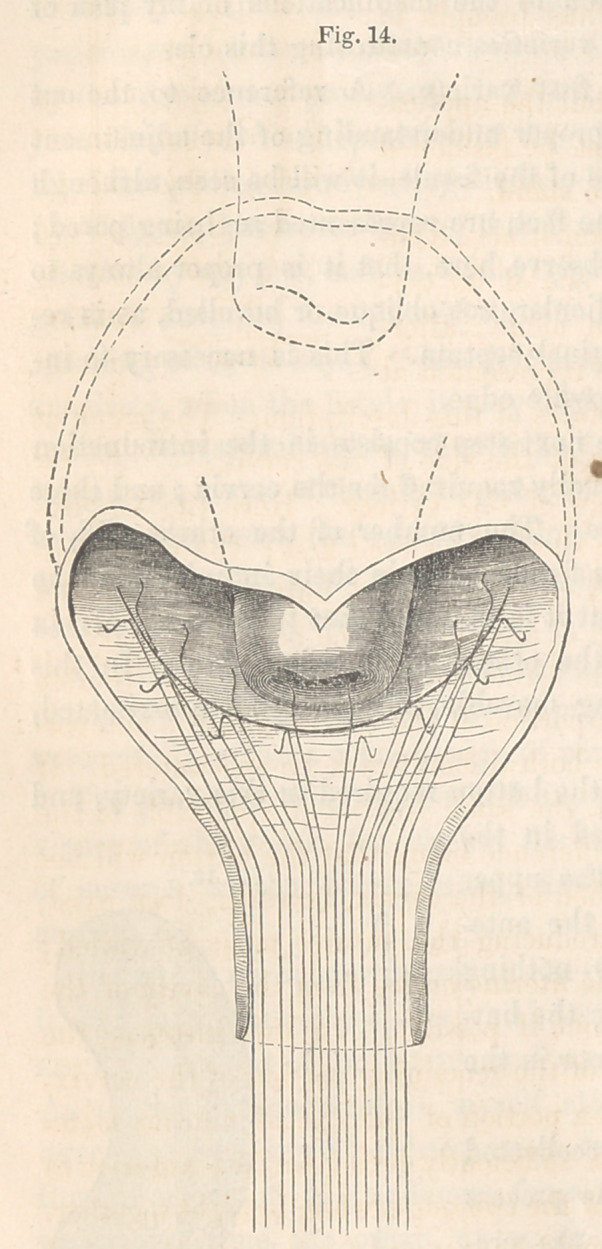


**Fig. 15. f15:**
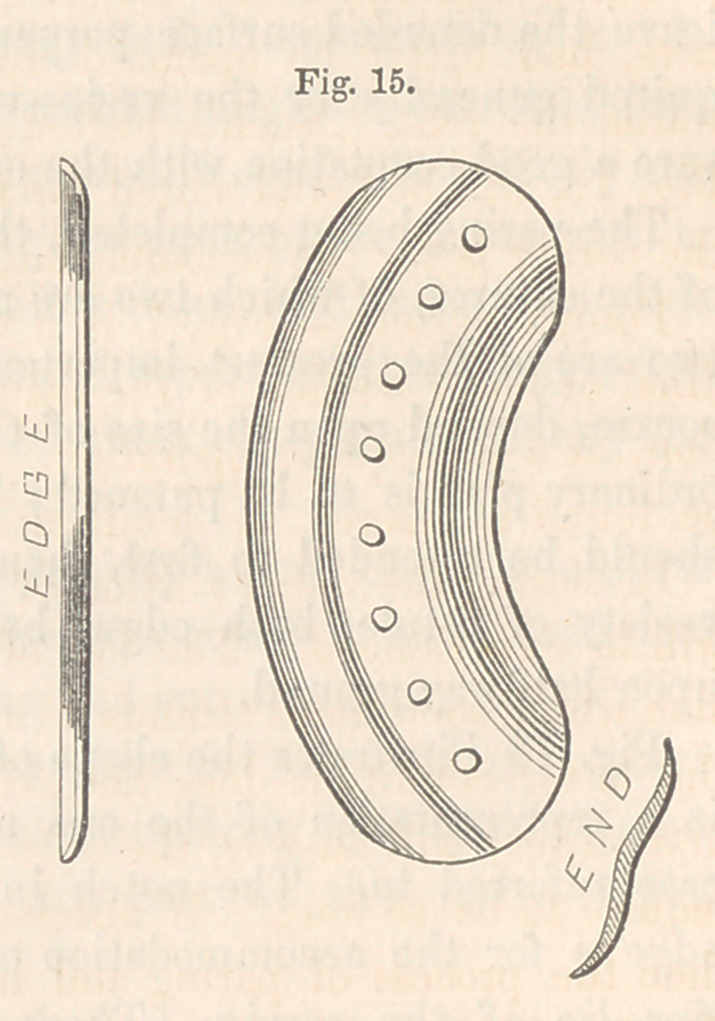


**Fig. 16. f16:**
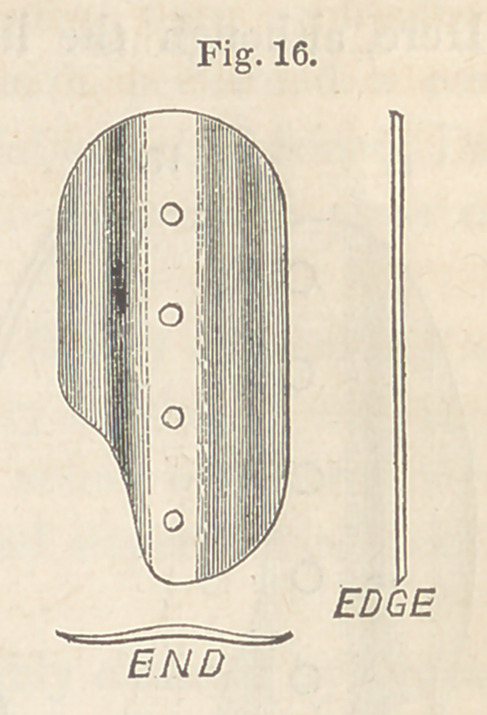


**Fig. 17. f17:**